# Collagen‐Based Scaffolds for Meniscal Repair and Regeneration

**DOI:** 10.1155/term/3446671

**Published:** 2025-12-31

**Authors:** Yizhuo Wang, Jenny Shepherd

**Affiliations:** ^1^ School of Engineering, University of Leicester, Leicestershire, Leicester, UK, le.ac.uk

**Keywords:** collagen scaffolds, meniscal repair, tissue engineering

## Abstract

Meniscal injuries present a significant clinical challenge due to the limited self‐healing capacity of avascular regions and the unsatisfactory long‐term outcomes of current repair strategies. Collagen, the primary structural component of the meniscal extracellular matrix (ECM), plays a crucial role in maintaining its biomechanical integrity and guiding tissue regeneration. This review summarizes recent advances in collagen scaffolds technology, focusing on materials, collagen extraction, and scaffold fabrication methods, as well as their in vivo interactions with cells that regulate tissue regeneration. The mechanical enhancement of collagen scaffolds through crosslinking and reinforcement with synthetic polymers is discussed, alongside strategies for controlled degradation and biological integration. Despite remaining challenges in mechanical durability and long‐term stability, these developments position collagen‐based scaffolds as a promising avenue toward clinically viable meniscal repair solutions.

## 1. Introduction

Meniscal tears are a common type of knee injury, affecting 60 to 70 individuals per 100,000 people each year. In the United Kingdom, approximately 70,000 people are hospitalized annually due to this injury, while in the United States, this figure is nearly five times higher [1]. Meniscal tears can be categorized by location and type and may occur in younger patients as a result of acute knee trauma or in older adults as part of the degenerative aging process. Acute meniscal tears frequently occur when a patient, with a flexed knee and planted foot, changes direction in a twisting or pivoting motion—an injury pattern common in sports with intense running and disguised movement. Degenerative meniscal tears, however, may occur with minimal trauma, as the meniscus becomes increasingly rigid and less compliant with age [2].

Due to the structural complexity of the meniscus and the variability of tear patterns, self‐repair of most meniscus injuries is not feasible. Arthroscopic partial meniscectomy (APM) involves the excision of damaged meniscal tissue, allowing for a smoother knee joint surface while preserving stable portions of the meniscus to maintain its function [3]. However, postoperative risks of osteoarthritis and cartilage degeneration have led to more conservative approaches in meniscus injury management. In a survey among surgeons, an increase in meniscal repair procedures was noted, with APM procedures reduced by 85%. National trends indicate a decrease in APM rates over the past two decades in countries such as France, Belgium, Germany, and Japan, alongside a substantial increase in repair rates [4].

The primary principle in meniscus surgery is the preservation of as much of the native meniscal tissue as possible [5]. Accordingly, there is a growing trend toward minimizing the use of APM and adopting regenerative approaches. One approach is the use of biodegradable scaffolds to promote meniscal regeneration, which is gaining increasing acceptance among both clinicians and the public. Given that collagen is the primary component of the meniscus’s extracellular matrix (ECM), this review will focus specifically on the advances, challenges, and future directions of collagen‐based scaffolds designed for meniscal repair and regeneration.

## 2. The Physiological Structure and Function of the Meniscus

The menisci are crescent‐shaped discs composed of fibrous cartilage, containing approximately 72% water, 22% collagen (primarily Type I), and 0.8% glycosaminoglycans (GAGs), mainly in the form of chondroitin‐6‐sulfate [6]. These components are produced and maintained by those fibroblast‐like cells on the edge, while fibrochondrocytes are responsible for their synthesis in the central regions. Serving multiple roles, the menisci are designed to withstand compressive forces, with the interplay between their structural elements enabling them to manage these stresses effectively [7]. Additionally, the menisci possess biomechanical properties crucial for enduring the various stresses exerted on them during the knee’s six degrees of freedom. The menisci can fit effectively against the femoral condyles and tibial plateau, which increases the stressed area of contact within the knee joint and helps protect the articular cartilage [8]. Their role is vital for load distribution and enhancing knee stability, especially in cases where the anterior cruciate ligament is deficient [9].

**Figure 1:**
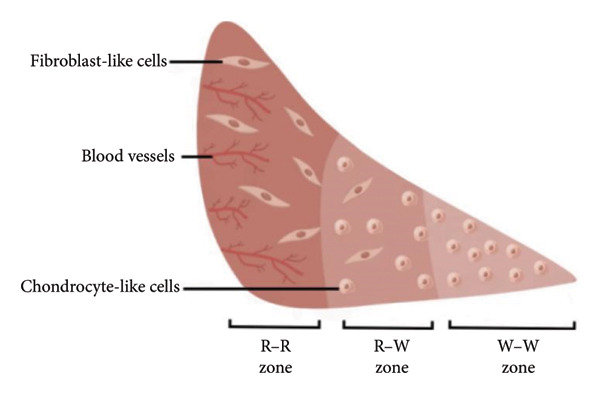
The cross‐sectional diagram of the meniscus with three main regions (by Figdraw).

As illustrated in Figure 1, the meniscus is divided into three zones based on vascularity: the outer red–red zone with good blood supply, the middle red–white zone with limited blood supply, and the inner white–white zone which is avascular [10]. The composition of the ECM in each zone varies, although types of collagen form the major constituent throughout. In the vascular zone (red–red zone), Type I collagen is the predominant type, constituting approximately 80% of the tissue’s dry weight. Conversely, in the avascular zone (white–white zone), Type I collagen accounts for 40% of the tissue’s dry weight, while Type II collagen makes up the remaining 60% [11].

## 3. Common Causes and Current Clinical Treatment Options for Meniscus Injuries

**Figure 2:**
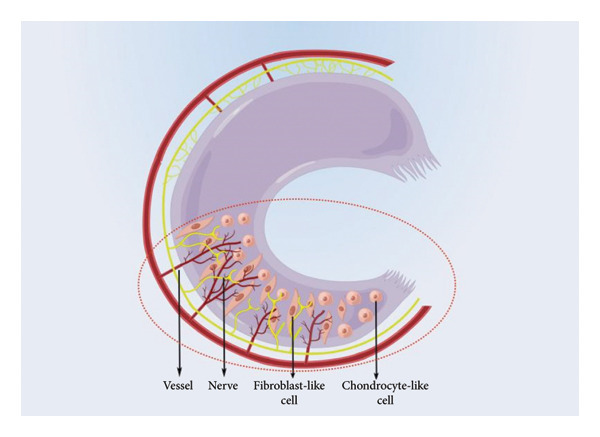
The distribution diagram of nerves, vessels, and cells within the meniscus (by Figdraw).

The cellular characteristics of the meniscus vary between its different zones. As shown in Figure 2, cells within the red–red zone display a fibroblast‐like morphology and are interconnected through cellular networks. In contrast, cells in the red–white zone and white–white zone exhibit a chondrocyte‐like phenotype and exist as individual cells. Adequate mechanical loading is crucial for preserving the structural integrity of the meniscus, as the lack of such loading may result in the derepression of catabolic genes and potential tissue atrophy [12]. Consequently, inadequate loading following an injury can contribute to the gradual deterioration of the affected tissue.

The variation in blood supply and cell distribution is crucial for understanding the healing potential of different regions of the meniscus. The limited blood supply to the meniscus makes healing after an injury especially challenging [13].

This limited healing capacity highlights the importance of understanding the nature of meniscal injuries, an injury with a propensity to occur in the young, fit, and economically active. Meniscal injury can lead to joint pain, swelling, and long‐term degenerative changes such as osteoarthritis if not properly managed [14]. These injuries typically result from a combination of axial loading and rotational forces applied to the knee. The severity of a meniscal tear can vary, ranging from minor tears that may heal on their own to complex tears that disrupt the structural integrity of the meniscus. According to the mechanism of injury, meniscal injuries can be classified as Transverse, Oblique, Longitudinal, Bucket handle, and Flap tears. The location of the tear is significant, and tears in the vascular zones have a higher potential for healing due to better blood supply, while tears in the avascular zones often fail to heal and may lead to further joint degeneration [15].

Traditional treatments for meniscal injuries include conservative management with physical therapy, APM, and meniscal repair. APM is considered a more conservative surgical alternative to total meniscectomy. It involves removing only the damaged portion of the meniscus to relieve symptoms, rather than excising the entire meniscus, which would increase mechanical stress on the articular cartilage. Nevertheless, the increase in localized contact stress peaks is widely recognized as a contributing factor to meniscal tissue degradation [16]. Given the meniscus’s protective function for tibial cartilage, this loss significantly raises the likelihood of joint cartilage degeneration and the subsequent need for arthroplasty [17]. While APM tends to yield more favorable outcomes due to the preservation of more tissue [18], it still carries a risk of complications commonly observed in meniscectomy procedures.

Meniscal repair preserves more of the meniscus tissue, and its biomechanical function to restore the natural structure and function of the meniscus is often desirable. However, the success of meniscal repair is limited by the meniscus’s poor self‐healing capacity, especially in the avascular white–white zone. This inherent limitation emphasizes the need for advanced scaffolds biomaterials designed to integrate with preserved tissue and facilitate functional regeneration.

Meniscal allograft transplantation (MAT) serves as a remedial approach to complications arising from meniscectomy. However, MAT seeks a minimal residual of native meniscal tissue, which requires removal of any remaining portions of the original meniscus prior to graft insertion [19]. For patients who have undergone APM and still have a peripheral rim for attachment, meniscal scaffolds offer an alternative treatment option [20]. Therefore, meniscal scaffolds play a crucial role in complementing meniscal scaffold implants.

Over the past three decades, substantial progress has been achieved in creating degradable meniscus scaffolds using both naturally derived materials and synthetic polymers. Notably, CMI (Stryker Corporation, Portage, MI, USA) [21] and Actifit (Orteq Sports Medicine Ltd., London, UK) [22] have obtained certification in Europe and are already being utilized in clinical settings. Nonetheless, these degradable scaffolds continue to face critical limitations. The newly generated tissue often resembles the meniscus but lacks the physiological structure and biomechanical traits of the native meniscus, making it insufficient for maintaining long‐term functionality of the knee joint or effectively safeguarding the articular cartilage.

As a naturally derived polymer, Type I collagen has been firmly established as a safe, biodegradable, and biologically compatible biomaterial, with several successful clinical trials demonstrating its potential in regenerative medicine [23].

The CMI and Actifit represent the two prominent types of meniscal scaffolds that are commercially available: biological scaffolds and synthetic polymer scaffolds. The CMI is a biologic scaffold composed of Type I collagen derived from bovine Achilles tendons, supplemented with hyaluronic acid (HA) and chondroitin sulfate. Preclinical studies demonstrated its capacity to support fibrochondrocyte infiltration, and histological analyses confirmed gradual resorption through cellular pathways including multinucleated giant cells [24, 25]. However, the mechanical properties of CMI remain suboptimal for maintaining normal knee joint function, with tensile stiffness reaching only approximately 40% of ovine meniscal tissue and suture pull‐out strength around 20 N, thereby restricting its use to cases with intact peripheral rims [21].

By contrast, the synthetic Actifit scaffold, composed of a porous polycaprolactone (80%)–polyurethane (20%) blend, exhibits a highly porous and interconnected architecture and undergoes slow degradation over approximately five years [26]. Actifit exhibits relatively low initial mechanical stiffness compared to the native meniscus, with experimental and computational studies indicating inferior compressive and tensile moduli, although it can partially restore tibiofemoral contact mechanics after APM [27]. Suture pull‐out strength testing has demonstrated values around 50 N, superior to those of CMI, while the Actifit still lacks the circumferential tensile reinforcement characteristic of the native meniscus [27].

Clinical outcomes for both scaffolds demonstrate significant improvements in patient‐reported outcome measures (PROMs), such as Lysholm, IKDC, Tegner, and KOOS scores, which remain stable over 5–10 years follow‐up periods [28]. However, pooled analyses and meta‐analyses reveal no clinically relevant differences between CMI and Actifit, with Tegner activity levels and PROM trajectories largely overlapping [26]. In terms of implant survival, CMI exhibits a reported failure risk of approximately 6%–7%, while Actifit demonstrates somewhat higher variability, with failure rates ranging from 6% to over 30% and an average 5‐year survival rate of 87%–88% [26, 29]. Importantly, imaging studies frequently show incomplete scaffold maturation, particularly with Actifit, despite stable symptom relief, suggesting a discordance between structural remodeling and functional recovery [30].

Meanwhile, promising advancements have also been made with alternative meniscus grafts. For instance, NUsurface (Active Implants) has reached clinical use in Europe and Israel and is currently in clinical trials in the United States [31]. In a two‐year clinical study, patients were randomized to receive medial meniscus replacement (MMR). At one‐year follow‐up, the MMR group showed significant improvements in pain, daily function, and quality of life compared with nonsurgical care. However, excessive or abnormal loading can cause implant failure or dislocation [32]. Thus, NUsurface is considered a transitional option to postpone more invasive procedures such as repeat meniscectomy or total knee arthroplasty.

Overall, while CMI and Actifit represent key advances in meniscal scaffold development, their limited mechanical strength, modest clinical benefits, and lack of clear chondroprotection restrict their use to partial meniscal defects. These constructs offer temporary mechanical support by stabilizing the joint and redistributing loads but fail to replicate native meniscus biomechanics. The more recent NUsurface implant provides an alternative synthetic option with encouraging short‐term outcomes, yet challenges in durability and biocompatibility persist. Future progress will depend on hybrid scaffold designs with enhanced tensile reinforcement, biological integration, and collagen‐based architectures capable of restoring the structural and functional complexity of the native meniscus.

## 4. Collagen Scaffolds in Meniscus Regeneration

### 4.1. The Importance and Role of Collagen Scaffolds

Acting as a vital structural framework within the body, the ECM provides structural support to cells while also storing numerous essential extracellular molecules, which play a pivotal role in preserving normal signaling cascades and tissue functionality [33]. Consequently, the fabrication of scaffolds that can mimic the ECM environment is crucial for cartilage repair, ensuring that these constructs remain cell‐friendly and promote the complete restoration of damaged tissues.

Serving as the predominant protein in the ECM, collagen is pivotal in preserving both the functional and structural stability of this essential tissue framework. It exhibits a remarkable level of adaptability, undergoing constant remodeling to support normal physiological operations. Within humans and other mammals, collagen stands as the most abundant structural protein in the ECM, principally responsible for providing cohesive support throughout the body. Over 20 different collagen types have been characterized, reflecting its diversity. Fibrillar collagen, which constitutes over 90% of the total collagen in human tissues, forms elongated fibrils through the aggregation of collagen microfibrils into hierarchical collagen complexes, with fibril diameters varying according to the specific tissue or organ [34]. Moreover, the notable capacity of collagen for binding various molecules highlights collagen’s potential in applications, such as drug delivery, growth‐factor administration, and cell carriers [35].

### 4.2. Material Selection

In the context of meniscal repair with scaffolds, two primary approaches can be identified: firstly, the creation of a permanent prosthesis, and secondly, the development of a biodegradable scaffold that supports tissue ingrowth, prompting the body to regenerate the lost tissue. Table 1, Tables 2, and 3 in this section provide a detailed comparison of the advantages and disadvantages of resorbable natural polymers, resorbable synthetic polymers, and nonresorbable synthetic polymers. These tables focus on the materials employed to replace the resected portion of the meniscus.

**Table 1:** Comparison of advantages and disadvantages of resorbable natural polymers.

Materials	Target tissue/application	Advantages	Disadvantages
Purified Type I collagen [36]	Meniscal repair and fibrocartilage regeneration	1. Protection of articular cartilage [36] 2. Stimulation of meniscal regeneration [24] 3. Enhanced meniscal function	1. No significant protection of articular cartilage from damage [24] 2. No improvement in meniscus function in acute patients without prior surgery [37] 3. Insufficient biomechanical performance 4. Dependency on vascularized edges of native meniscus limits usability

Silk [38]	Meniscus and articular cartilage repair	1. Superior toughness [39] 2. Adjustable properties [40]	1. Initial mechanical properties require adjustment [41] 2. Increased silk fibroin concentration enhances mechanical properties but reduces porosity and interconnectivity [40]

Bacterial cellulose [42]	Cartilage and tendon tissue engineering	1. Cost‐effective 2. Promotes cell migration 3. Higher compression modulus	1. Low compression modulus 2. Lacks detailed material characterization

Decellularized extracellular matrix (dECM) [43, 44]	Meniscal tissue engineering and fibrocartilaginous scaffold design	1. Preserves native ECM composition and collagen fibrillar architecture 2. High biomimicry with zonal specificity 3. Promotes cell adhesion, differentiation, and matrix remodeling 4. Biodegradable and biocompatible	1. Batch‐to‐batch variability 2. Possible loss of bioactivity during decellularization 3. Insufficient mechanical strength 4. Risk of residual immunogenic components

**Table 2:** Comparison of advantages and disadvantages of resorbable synthetic polymers.

Materials	Advantages	Disadvantages
Polyurethanes [45]	1. Larger macropores (200–300 μm) with high interconnectivity and porosity promote cell and tissue ingrowth [46] 2. Delay fibrocartilage degradation and ingrowth [47]	Carbon particles cause synovitis

Estane 5701F [48]	1. Excellent processability and elasticity suitable for flexible scaffold fabrication	1. Poor mechanical properties 2. Decreased mechanical performance 3. Cannot prevent cartilage damage in joints

PCLPU [49]	1. Faster inward tissue growth [50] 2. Increased compression modulus	1. Cannot prevent cartilage damage in joints [51] 2. Cannot differentiate into natural meniscus tissue 3. Lack of specific collagen fiber orientation 4. Formation of harder tissue [52]

Actifit [22]	1. Increased contact area 2. Enhanced load distribution 3. Successful inward tissue growth [53]	1. Cannot return to normal knee joint levels 2. Poor initial friction behavior [54]

Porous composite of polycaprolactone (PCL) and HYAFF [55]	Reduced cartilage damage	1. Cartilage damage 2. Compression occurs 3. Irregular surface with wrinkles 4. Insufficient biomechanical properties 5. Difficult to fix

Blend of poly(L‐lactic acid) and poly(p‐dioxanone) [56]	1. Bioresorbable scaffold allowing tissue ingrowth 2. Induces fibrocartilage formation 3. Provides significant cartilage protection	Limited load‐bearing properties.

Poly(L‐co‐D,L‐lactic acid) (PLDLA) copolymer [57]	Enhanced degradation profile.	1. The methods for evaluating cartilage damage are limited. 2. Higher number of chondrocytes. 3. More complex in vivo experiments are needed.

Degradable tyrosine‐derived polymer [58]	1. Absorbable meniscus scaffold 2. The tensile load increases with the increase in fiber density	Further in vivo experiments are needed in large animal models.

**Table 3:** Comparison of advantages and disadvantages of nonresorbable synthetic polymers.

Materials	Advantages	Disadvantages
Teflon (DuPont) [59]	Low friction coefficient	1. Cannot prevent cartilage damage. 2. Mechanical and tribological properties are inconsistent with natural tissue.

Polyurethane‐coated Dacron felt prosthesis [60]	1. Permanent prosthesis 2. Outstanding crease and abrasion resistance. 3. Enhanced abrasion, tear, and impact resistance	1. Causes synovitis. 2. Cannot prevent cartilage damage.

Poly(vinyl alcohol) hydrogel (PVA‐H) [61]	1. Demonstrated biocompatibility 2. Mimicking human tissue 3. Lower modulus comparison [62]	1. Cannot prevent cartilage damage. 2. Insufficient mechanical properties.

Artificial polycarbonate–urethane [31]	1. Biostability and biocompatibility 2. Redistribute a load similar to that of a natural meniscus [63] 3. Flexible and easily deformable [64]	More effective experiments are needed.

Thermoplastic elastomer [65]	1. Anatomical design, nonabsorbable 2. Suitable for replacing severely damaged knee joints 3. Cartilage‐protective properties	Animal experiments are needed to evaluate the performance of the implant under long‐term load conditions and its cartilage‐protective ability.

Table 1 provides a comprehensive summary of the advantages and disadvantages of resorbable natural polymers commonly used in tissue engineering, including purified Type I collagen, silk, bacterial cellulose, and dECM. These materials target different tissues such as meniscus, cartilage, and tendon, reflecting their diverse structural and biological properties. Purified Type I collagen [24, 36, 37] supports meniscal regeneration and cartilage protection, but it is limited by insufficient mechanical strength and reliance on vascularized tissue edges. Silk [38–41] offers superior toughness and tunable degradation behavior, although optimization of stiffness and porosity remains necessary. Bacterial cellulose [42] is a cost‐effective material that promotes cell migration but lacks adequate tensile strength and detailed long‐term characterization. In contrast, dECM [43, 44] provides a collagen‐rich, biomimetic framework that preserves native extracellular composition and enhances cell–matrix interactions, yet suffers from variability and limited mechanical robustness. Overall, this comparison highlights the balance between biological fidelity and mechanical performance that must be considered when selecting appropriate scaffolds for specific clinical applications.

Table 2 summarizes the key properties of various resorbable synthetic polymers, emphasizing their potential and limitations in biomedical applications. Polyurethanes [45–47] promote cell and tissue ingrowth but may induce synovitis due to carbon particles. Estane 5701F [48] exhibits poor mechanical performance and fails to prevent cartilage damage. PCLPU [49–52] enables faster tissue growth and higher compression modulus but lacks collagen fiber orientation and differentiation potential. Actifit [22, 53, 54] enhances load distribution and tissue growth but has poor initial friction behavior and fails to restore normal knee joint functionality. Porous composites of PCL and HYAFF [55] reduce cartilage damage yet suffer from biomechanical weaknesses and irregular surfaces. Blends of poly(L‐lactic acid) and poly(p‐dioxanone) [56] provide cartilage protection but lack extensive follow‐up studies. Poly(L‐co‐D,L‐lactic acid) copolymers [57] and degradable tyrosine‐derived polymers [58] show promising degradation profiles and mechanical improvements but require further in vivo experimentation for broader clinical validation. This analysis highlights the critical trade‐offs in the development of synthetic polymer scaffolds for meniscal regeneration.

Nondegradable, permanent implants do not depend on tissue infiltration or decomposition. In this context, the absence of cytotoxicity, long‐term structural stability in vivo, and the ability to mimic the mechanical properties and tribological behavior of the native meniscus have become key requirements for such implants. These implants should distribute loads effectively throughout the knee joint and help prevent excessive localized stress. Representative examples of nonresorbable synthetic polymers used for meniscal replacement, along with their advantages and disadvantages, are summarized in Table 3.

In summary, the selection of materials for meniscal regeneration involves a careful consideration of both the biological and mechanical properties of the chosen scaffold. While resorbable natural and synthetic polymers offer promising advantages in terms of biocompatibility, tissue integration, and biodegradability, they are often limited by trade‐offs in mechanical performance, degradation rates, and tissue‐specific functionality. The development of nondegradable permanent implants, while sidestepping issues of degradation and tissue ingrowth, requires ensuring long‐term stability and biocompatibility to closely mimic the native meniscus in terms of both mechanical load transfer and tribological behavior. Ultimately, the advancement of meniscal repair strategies will depend on the continued refinement of material properties, as well as the integration of these materials into effective, functional scaffolds capable of supporting long‐term joint health and functionality.

### 4.3. Construction of Collagen Scaffolds

**Figure 3:**
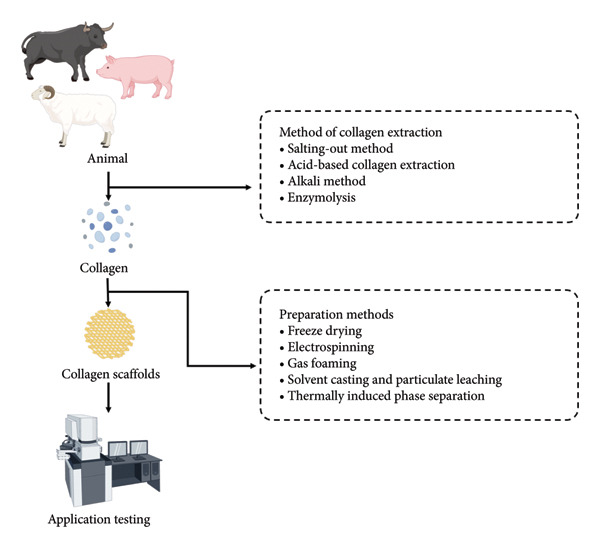
Collagen scaffold production process (by Figdraw).

Collagen functions as the major fibrillar component of the meniscus with multiple collagen types occurring in different proportions throughout the tissue. Types I and II dominate, with Type I widely distributed across most of the meniscus, whereas Type II is chiefly localized in its central region [66]. The collagen fiber orientation endows the meniscus with its load‐bearing properties [67]. Figure 3 demonstrates the procedure for creating a collagen scaffold, outlining the major production stages.

The first stage in collagen‐based scaffold fabrication is the extraction of collagen from native tissues, typically involving pretreatment, extraction, and purification. The choice of collagen source significantly affects product safety and yield, as animal‐derived collagen may carry risks of pathogen transmission [68]. Therefore, careful selection of collagen sources is essential to ensure the safety and efficacy of biomedical scaffolds.

Table 4 summarizes the different methods of collagen extraction. Each method has unique advantages and limitations that make it suitable for specific applications. The salting‐out method provides a high collagen recovery rate without disrupting its triple‐helix structure, but it requires proper pretreatment to ensure effective protein precipitation. Acid‐based extraction, which uses organic acids such as acetic and citric acid, excels at solubilizing non‐crosslinked collagen and breaking interstrand crosslinks, thus increasing the yield of soluble collagen. In contrast, the alkali method preserves collagen’s bioadhesive properties and achieves high recovery but may weaken fibril formation and cause some amino acid degradation. Enzymolysis, often paired with acid‐based extraction, offers precise control over collagen hydrolysis while limiting protein damage, but it is costlier and takes longer. Each approach underscores the balance between extraction efficiency, yield, and maintaining the structural integrity of collagen.

**Table 4:** Method of collagen extraction.

Method	Common reagent	Typical sources	Advantage	Disadvantage
Salting‐out method [69]	Sodium chloride (NaCl), tris(hydroxymethyl)aminomethane hydrochloride acid (Tris‐HCl), and citrate	Bovine Achilles tendon, porcine skin, and meniscal tissue from porcine knee joints	1. High collagen recovery rate. 2. The integrity of the triple‐helix structure.	Improper preprocessing of raw materials may hinder protein precipitation.

Acid‐based collagen extraction [70]	Organic acids	Bovine dermis, fish skin, bovine meniscus	Organic acids can solubilize non‐crosslinked collagen and disrupt intermolecular crosslinks, thereby increasing the yield of soluble collagen [71].	Prolonged acid exposure may partially denature collagen and reduce fibrillogenesis capacity.

Alkali method [72]	Sodium hydroxide (NaOH)	Porcine skin, bovine tendon, cartilage	1. High recovery of collagen. 2. Maintaining its ability to support the adhesion of human keratinocytes and fibroblasts. 3. The substantial yield of collagen protein.	1. Collagen can no longer form fibrils under physiological conditions with a neutral pH 2. This process may damage amino acids that contain hydroxyl or sulfhydryl groups.

Enzymolysis [73]	Pepsin, tryptase, and papain	Fish skin, bovine tendon, porcine meniscus ECM	1. Boost collagen yield. 2. Reduce the reaction duration. 3. It allows for precise control over the extent of hydrolysis. 4. Gentler on the collagen protein. 5. The lower overall salt content.	1. Relatively costly. 2. The reaction duration may increase depending on the specific enzyme employed.

The biosynthesis and hierarchical assembly of collagen are well understood, providing a foundation for tailoring scaffold fabrication processes to specific tissue engineering needs [74]. Freeze‐drying is a widely adopted method for fabricating collagen scaffolds due to its mild processing conditions, which preserve the protein’s native structure and bioactivity [75]. This technique allows the solvent to be removed by sublimation under controlled temperature and vacuum conditions, forming an interconnected porous network. The morphology and pore size of the resulting scaffold can be precisely tuned by adjusting the freezing rate, temperature gradient, and sublimation time [76]. Rapid freezing typically produces small, uniformly distributed pores, while slower freezing creates larger, anisotropic structures, enabling researchers to tailor scaffold microarchitecture for specific biological functions. These freeze‐dried scaffolds exhibit excellent biocompatibility and mechanical stability, providing an ideal environment for cell adhesion, migration, and matrix deposition [77].

In recent years, the integration of freeze‐drying with 3D printing technologies has opened new possibilities for the fabrication of complex collagen‐based constructs. Compared with conventional scaffold fabrication methods, 3D printing offers superior reproducibility and precise control over both macro‐ and microscale architectures. Low‐temperature deposition 3D printing, in particular, enables the co‐printing of biodegradable polymers such as PCL with collagen solutions, ensuring the preservation of collagen’s triple‐helical structure and biological functionality. This hybrid approach allows the creation of multilayered scaffolds with spatially controlled porosity and compositional gradients that mimic native meniscal or osteochondral tissue organization [78]. Moreover, incorporating bioactive agents or living cells into collagen‐based bioinks during printing further enhances the scaffold’s regenerative potential. Overall, these advances highlight the synergistic potential of freeze‐drying and 3D printing in producing biomimetic, structurally precise, and biologically active collagen scaffolds for next‐generation tissue engineering applications.

### 4.4. Cell Recruitment and Maturation in Collagen Scaffolds

The regenerative success of collagen‐based scaffolds relies not only on their structural fidelity but also on their capacity to modulate cell recruitment, adhesion, and maturation. Collagen inherently presents cell‐binding motifs such as arginine–glycine–aspartic acid (RGD) sequences, which facilitate integrin‐mediated adhesion, triggering downstream signaling to regulate cytoskeletal arrangement and ECM synthesis. The high porosity and interconnectivity of well‐fabricated collagen scaffolds also support nutrient diffusion and cellular infiltration, establishing a microenvironment favorable for cell colonization and remodeling.

In the early phase postimplantation, collagen scaffolds can recruit endogenous cells including fibrochondrocytes, synovial mesenchymal stem cells (MSCs), and bone marrow–derived MSCs from adjacent tissues and fluid compartments. The gradual degradation of collagen may release bioactive peptides that serve as chemoattractants, enhancing cell migration and promoting angiogenesis and scaffold remodeling. For example, meniscus‐derived matrix scaffolds have been shown to permit infiltration of endogenous meniscal cells and MSCs, promoting integrative repair in vitro and in vivo models [79]. In a meniscal context, MSC–scaffold interactions have been demonstrated to yield enhanced Type I/II collagen and GAG deposition, which more closely mimics native meniscal ECM [80].

Once seeded or recruited, cells within the scaffold undergo proliferation, ECM production, and phenotypic maturation. The alignment of collagen fibers can direct cell orientation and anisotropic ECM deposition consistent with physiological load paths. Biochemical cues, such as incorporated growth factors or residual dECM signals, further drive chondrogenic differentiation, leading to deposition of regionally appropriate collagen types and proteoglycans, which is a key advantage of biomimetic scaffolds [80].

An emerging strategy is to couple mechanical stimulation with scaffold design to accelerate maturation. Dynamic loading regimes such as cyclic compression, hydrostatic pressure, or tensile strain have been shown to upregulate ECM gene expression, align collagen fibers, and enhance the mechanical competence of engineered constructs. A recent study on fibroblasts within microchanneled scaffolds demonstrated that mechanical stimulation boosts oriented collagen fiber production [81]. Another work showed that controlled mechanobiological cues can significantly speed up ECM deposition and tissue maturation [82]. However, mechanical cues must be optimized carefully, as excessive loading may impair cell viability or lead to over‐remodeling [83].

In summary, collagen scaffolds serve not only as passive structural templates but as active modulators of cell recruitment and maturation. The interplay of biochemical motifs, scaffold architecture, controlled degradation, and mechanical stimulation dictates the success of new tissue formation. Achieving synchronized scaffold resorption and neotissue formation is critical: The scaffold must support early cell ingress and matrix deposition, then gradually yield to endogenous matrix, culminating in a fully functional, load‐bearing neomeniscus.

## 5. Strategies to Achieve Native Biomechanical Properties

### 5.1. The Biomechanical Challenge

Successful meniscal repair requires not only biological integration but also the restoration of native biomechanical function. The meniscus principally distributes load between the femoral and tibial articular surfaces, bearing 50%–70% of the weight during load‐bearing activities [84]. Establishing quantitative mechanical benchmarks from the natural meniscus is therefore essential for defining design targets for collagen‐based scaffolds. The native meniscus displays pronounced anisotropy, with collagen fibers organized circumferentially to resist hoop stresses while radial tie fibers prevent splitting under compression and shear [85–87].

Human meniscal tissue exhibits circumferential tensile moduli typically between 80 and 160 MPa and ultimate tensile strengths (UTS) of 10–25 MPa, depending on region and orientation [86]. In contrast, radial moduli are one to two orders of magnitude lower (1–6 MPa) [88]. These properties enable efficient stress dissipation and joint stability under physiological loading. To recapitulate these native biomechanical characteristics, an ideal meniscal scaffold should exhibit regionally graduated mechanical properties comparable to those of the native meniscus. Achieving such target values would better reproduce the anisotropic load‐bearing behavior of the native meniscus and minimize implant deformation or failure under dynamic joint loading.

However, pure collagen scaffolds such as CMI remain mechanically inferior, achieving only 20%–40% of the circumferential stiffness of native meniscus and exhibiting UTS values often below 1 MPa [89, 90]. The weak, randomly oriented collagen network lacks the hierarchical alignment and dense crosslinking of the natural fibrocartilaginous matrix. Furthermore, degradation during early remodeling further reduces load‐bearing capability, preventing long‐term maintenance of hoop stress and joint stability.

Addressing this gap requires hybrid scaffolds capable of reproducing the anisotropic fiber orientation and viscoelastic behavior of the native meniscus. Recent approaches include circumferential fiber reinforcement, composite polymer–collagen networks, and guided mechanical remodeling [81, 91]. In addition, chemical crosslinking offers a complementary route to stabilize collagen’s structure and enhance its strength, collectively advancing these scaffolds toward the mechanical performance of native meniscal tissue.

### 5.2. Strategies for Mechanical Enhancement

Although collagen scaffolds possess excellent biocompatibility, their poor intrinsic mechanical strength and rapid enzymatic degradation limit their clinical durability. To address these shortcomings, mechanical enhancement strategies have focused on crosslinking and the development of hybrid or fiber‐reinforced scaffolds that combine collagen with stronger synthetic polymers.

Physical crosslinking relies on modifying collagen scaffolds via processes like dehydrothermal (DHT) [92] treatment, ultraviolet (UV) exposure, or gamma and electron beam irradiation. For instance, DHT has been shown to boost the degree of crosslinking as temperatures rise, thereby tripling the tensile strength of collagen films compared to their untreated counterparts [93]. Meanwhile, UV, gamma, and electron beam treatments generate intermolecular linkages through free radical formation [94]. These irradiation methods also serve as common sterilization approaches and can be paired with other crosslinking strategies. Although UV irradiation minimally disrupts cells, on its own, it does not exhibit a notably high crosslinking density [95].

Chemical crosslinking involves the use of specific reagents to create covalent bonds among collagen molecules or collagen‐based biomaterials, thus boosting mechanical resilience, slowing degradation, and reinforcing overall structure. Typically, this entails incorporating functional groups—carboxyl, amino, or hydroxyl—into collagen so that crosslinks can form. One widely adopted technique is carbodiimide‐mediated crosslinking, which utilizes EDC (1‐ethyl‐3‐(3‐dimethylaminopropyl) carbodiimide) and NHS (N‐hydroxysuccinimide) to encourage amide bond formation between amino and carboxyl groups in collagen [96]. By enhancing the mechanical behavior of collagen scaffolds, this approach makes them more suitable for applications in tissue engineering and regenerative medicine. However, these treatments provide only moderate improvements in tensile modulus and may reduce flexibility when excessive crosslinks are formed. Consequently, crosslinking alone is insufficient to achieve the tensile strength and anisotropic stiffness characteristic of the native meniscus.

A more effective strategy involves hybrid collagen–polymer scaffolds, which integrate collagen with resorbable synthetic polymers such as PCL, polylactic acid (PLA), or silk fibroin. These composites offer improved stiffness, suture retention, and fatigue resistance while maintaining biodegradability. Merriam et al. [97] demonstrated that embedding PCL fibers within a Type I collagen matrix increased tensile strength by nearly an order of magnitude. Similarly, Gao et al. [98] developed aligned PCL–collagen hybrids achieving a circumferential tensile modulus of 8.5 ± 1.9 MPa and 2.3 ± 0.3 MPa in the crosswise direction. Incorporating silk fibroin further enhances toughness and elastic recovery, as reported by Duan et al. [99], without compromising cellular compatibility.

Recent progress in fiber‐reinforced and 3D‐printed scaffolds enables precise spatial organization of collagen and synthetic components. Patel et al. [92] showed that polymer fibers oriented circumferentially within collagen matrices significantly improved hoop strength and sustained load‐bearing capacity after implantation. Additive manufacturing provides reproducible control over scaffold geometry and fiber orientation, allowing PCL or silk microfilaments to be deposited in architectures mimicking native circumferential alignment [99]. Low‐temperature and coaxial extrusion methods preserve collagen’s bioactivity while embedding reinforcing filaments that absorb most mechanical stress. These 3D‐printed hybrid constructs exhibit elastic moduli and UTSs several times greater than pure collagen scaffolds, substantially narrowing the mechanical gap with native tissue.

Collectively, these hybridization and reinforcement approaches have transformed collagen scaffolds from soft, resorbable matrices into load‐bearing biomaterials capable of withstanding joint‐level stresses. Future efforts integrating multimaterial printing, graded reinforcement, and biologically driven remodeling cues are expected to yield scaffolds that replicate both the composition and mechanical anisotropy of the native meniscus [99–101].

### 5.3. Mimicking Native Architecture

Replicating the intricate architecture of the native meniscus is essential for achieving both mechanical and biological fidelity. Beyond compositional similarity, scaffold performance is governed by microstructural parameters, such as porosity, pore interconnectivity, overall form, and fiber alignment. The meniscus exhibits highly organized collagen bundles oriented predominantly in the circumferential direction, which confer anisotropic tensile strength and enable conversion of compressive loads into hoop stresses. Therefore, scaffolds that fail to reproduce this anisotropic organization cannot fully restore native biomechanics or long‐term functionality, as homogeneous scaffolds have been shown to lead to joint degeneration [102]. Advanced approaches incorporating aligned fiber architectures [98] and stem cell–based strategies [80] have been developed to address this critical challenge.

Different scaffold morphologies offer distinct advantages but also inherent limitations when applied to meniscal repair. Hydrogels, though biocompatible and easily loaded with cells or growth factors, possess weak mechanical integrity and isotropic networks that cannot sustain complex multiaxial loads typical of the knee joint. Sponge‐type collagen scaffolds, such as freeze‐dried collagen, provide interconnected porosity for cell infiltration but lack the fiber orientation necessary for directional stress transfer. Membrane or film scaffolds exhibit higher in‐plane stiffness yet remain too thin and structurally homogeneous to bear circumferential tension or resist compressive deformation. As a result, these conventional forms are often restricted to partial defect filling or serve only as temporary matrices rather than functional load‐bearing replacements [103].

To overcome these limitations, recent work has emphasized anisotropic and fiber‐aligned scaffold architectures. Techniques such as electrospinning, melt electrowriting, and low‐temperature 3D printing enable the controlled deposition of collagen or composite microfibers along predefined directions, reproducing the circumferential and radial fiber networks of the native meniscus. Aligned fiber arrays significantly increase tensile stiffness and suture retention strength, achieving up to 3‐ to 7‐fold improvements compared with randomly oriented counterparts [104]. Moreover, the anisotropic microstructure directs cell elongation and ECM deposition along load‐bearing axes, promoting the formation of mechanically functional neotissue. Studies using hybrid PCL–collagen and silk–collagen scaffolds have demonstrated that controlled fiber alignment enhances fibrochondrogenic differentiation and leads to more uniform Type I/II collagen distribution within regenerated constructs [99].

In this context, porosity and anisotropy must be co‐optimized. High porosity (> 70%) is needed for nutrient transport and cellular infiltration, yet excessive pore size reduces load capacity. Scaffold designs that integrate multiscale porosity with aligned fibrous frameworks have shown superior mechanical efficiency and biological performance [105]. The next generation of biomimetic meniscal scaffolds will employ gradient or hierarchical architectures, combining soft, hydrated matrices with stiff, circumferential fiber reinforcement to recapitulate the region‐specific anisotropy of the native meniscus. Such designs represent a decisive step toward functional regeneration capable of enduring physiological joint loads. Furthermore, promoting region‐specific matrix deposition, particularly enhanced proteoglycan accumulation in the inner zone and aligned collagen organization in the outer zone, is essential for recapitulating the native compositional and functional heterogeneity of the meniscus.

## 6. Long‐Term Clinical Translation and Remaining Bottlenecks

### 6.1. Critical Evaluation of Clinical Outcomes

Failure analyses indicate that both CMI and Actifit are prone to mechanical fatigue, partial resorption, and inflammatory reactions, which can lead to revision or conversion to arthroplasty in about 6% to 30% of cases within five to ten years [106]. These failures are often associated with insufficient scaffold stiffness and loss of circumferential integrity during repetitive joint motion. Moreover, the success of collagen‐based scaffolds, especially CMI, is largely dependent on the presence of a vascularized peripheral rim, which provides essential nutrient supply and anchorage for cellular ingrowth. In defects extending into the avascular white–white zone, tissue regeneration remains poor and integration is incomplete. This vascular dependency fundamentally limits the indication of such scaffolds to partial meniscal replacements where peripheral attachment is preserved.

When compared with MAT, both CMI and Actifit show lower long‐term survival and weaker chondroprotective effects. MAT generally achieves graft survivorship of about 80% at ten years with moderate preservation of cartilage, whereas scaffold‐based reconstruction tends to decline in performance after five to seven years, often with signs of progressive cartilage wear on follow‐up imaging [107–109]. However, MAT is limited by donor availability, potential immune response, and surgical complexity, which maintains the appeal of scaffolds as a less invasive alternative despite their mechanical limitations and vascularization constraints.

At present, none of the available implants have demonstrated consistent evidence of cartilage preservation or sustained joint protection. The absence of randomized comparative trials between scaffolds and MAT further complicates clinical interpretation. Biodegradable meniscal scaffolds can provide symptom relief and partial functional improvement in the short term, but their durability, integration, and cartilage‐preserving potential remain uncertain. Their clinical success is also fundamentally limited by their reliance on vascularized peripheral rims, which restricts effective regeneration in the central avascular zone. Further progress will depend on the development of mechanically reinforced and biologically integrative scaffolds capable of supporting cellular ingrowth and remodeling even in avascular environments, validated through standardized, multicenter clinical trials with extended follow‐up.

### 6.2. The Bottlenecks for Collagen Scaffolds

Despite steady progress in biomaterial design and clinical research, collagen‐based meniscal scaffolds are still far from routine clinical application. Several intrinsic and manufacturing‐related challenges continue to limit their long‐term reliability and broad clinical use.

The first and most evident limitation lies in their insufficient mechanical strength. The native meniscus exhibits tensile moduli in the range of 80 to 160 MPa in the circumferential direction, enabling it to withstand repetitive compressive and shear loads within the knee joint. In comparison, collagen scaffolds rarely exceed 10 to 30 MPa even after chemical crosslinking or polymer reinforcement [97, 98]. Their random fibrillar organization and lack of circumferential alignment result in poor load transfer, limited suture retention, and structural fatigue under repetitive motion. This mechanical inferiority remains one of the primary reasons for incomplete tissue regeneration and implant failure in clinical use.

Another major challenge concerns the control of degradation kinetics. Collagen’s natural biodegradability, although advantageous for remodeling, becomes problematic when the rate of enzymatic breakdown exceeds the pace of new matrix formation. Rapid degradation can lead to early loss of mechanical support, whereas excessive stabilization by crosslinking may inhibit cell infiltration and remodeling [92, 110]. Achieving a balanced and predictable degradation profile therefore remains a key objective, particularly for scaffolds intended to function over several months before full resorption.

Batch‐to‐batch variability also represents a significant obstacle to reproducible performance. Collagen derived from bovine or porcine sources often exhibits differences in fibril diameter, crosslink density, and impurity levels, all of which influence mechanical behavior and host response [111]. Even small deviations in extraction or sterilization protocols can alter fibrillar integrity and modify immunogenic potential. The absence of strict standardization complicates quality control, regulatory approval, and large‐scale clinical production.

Finally, the long‐term biological stability of collagen scaffolds remains limited. Progressive hydrolytic and enzymatic degradation, combined with low‐grade inflammatory remodeling, can result in shrinkage, decreased elasticity, and inconsistent collagen fiber reorganization over time [7]. Clinical follow‐up studies often reveal partial resorption without complete mechanical or structural recovery, suggesting that persistence of the scaffold does not equate to sustained function. Improving long‐term stability will require advanced crosslinking strategies, hybrid polymer reinforcement, and modulation of host–material interactions to prevent excessive degradation.

In summary, the clinical translation of collagen scaffolds continues to be restricted by low mechanical strength, difficulty in achieving controlled degradation, batch‐to‐batch variation, and limited biological stability in long‐term use. Addressing these bottlenecks demands an integrated approach that unites molecular‐level stabilization with macroscale architectural design and standardized manufacturing, enabling the creation of collagen‐based scaffolds that are both mechanically durable and biologically reliable for meniscal regeneration.

## 7. Future Research Directions

### 7.1. Achieving Anisotropy and Compositional Gradients

Replicating the meniscus’s structural hierarchy requires not only reproducing its biochemical composition but also capturing its pronounced anisotropy and zonal gradients. The native meniscus contains circumferentially aligned collagen bundles that bear hoop stresses and a radial network that resists splitting, while its composition transitions gradually from the fibrocartilaginous red–red zone rich in Type I collagen to the more cartilaginous white–white zone dominated by Type II collagen and proteoglycans. Mimicking both fiber orientation and compositional variation is therefore a central objective for next‐generation collagen‐based scaffolds [98, 112].

Recent advances in biofabrication and additive manufacturing have made this goal increasingly feasible. High‐precision methods such as melt electrowriting (MEW) and low‐temperature extrusion printing allow controlled deposition of micro‐ and nanofibers along predefined toolpaths. By aligning collagen or composite filaments circumferentially, these techniques generate anisotropic scaffolds that reproduce the load‐bearing directionality of native tissue. Studies have shown that MEW‐printed PLLA lattices can achieve tensile strength approaching 66.6 MPa, guiding cell elongation and ECM deposition along fiber axes [113]. Similarly, shear‐induced alignment during extrusion bioprinting promotes the formation of unidirectional collagen fibrils within each filament, resulting in improved stiffness and oriented cellular morphology without compromising cell viability [114].

Equally important is the recreation of biochemical zonation from the vascularized red–red region to the avascular white–white region. Multimaterial and gradient bioprinting enable controlled spatial variation in composition, permitting a gradual transition from Type I–dominant collagen matrices near the periphery to Type II–rich, proteoglycan‐laden regions in the center. Several groups have implemented sequential or coaxial printing strategies to deposit collagen blends with distinct ratios of Type I and Type II, or to incorporate dECM fractions representing outer and inner meniscal zones [102]. These compositional gradients not only replicate native biochemical heterogeneity but also modulate local cell phenotypes, promoting fibrochondrogenic differentiation in a spatially organized manner.

Integration of anisotropy and gradient control represents the frontier of meniscal scaffold design. By combining aligned fiber deposition with region‐specific bioinks, researchers are now producing constructs that closely approximate the native meniscus in both architecture and composition [115]. Postprinting crosslinking and controlled mineral or GAG loading further allow fine‐tuning of local stiffness and degradation. Collectively, these strategies define a new generation of biofabricated collagen scaffolds capable of emulating the hierarchical fiber orientation and compositional transitions of the meniscus, providing a realistic path toward fully functional, load‐bearing regeneration.

One of the key challenges in collagen‐based meniscus scaffolds is replicating the mechanical properties and degradation profile of native meniscal tissue. To address this, incorporating bioactive fillers such as hydroxyapatite or calcium phosphate into collagen matrices shows potential for enhancing mechanical strength, stiffness, and load‐bearing capacity while maintaining biocompatibility. Advances in crosslinking methods, including the use of glutaraldehyde, EDC/NHS, genipin, and natural crosslinkers, have demonstrated improvements in tensile strength and deformation resistance. However, careful optimization of crosslinking levels is essential to avoid compromising biological functionality. Additionally, fabrication technologies like 3D printing offer unprecedented control over scaffold architecture, allowing for the design of structures with tailored porosity and mechanical properties. Future research should aim to fully replicate the anisotropic mechanical characteristics of native meniscal tissue, enabling scaffolds to endure physiological loads more effectively. This involves exploring multimaterial 3D printing and gradient crosslinking techniques to achieve a seamless integration of strength and flexibility.

### 7.2. Bioactive Factor and Stem Cell Delivery Systems

The degradation rate of collagen scaffolds is critical for tissue engineering applications, as it must align with the rate of regeneration of new tissue. Future studies should investigate the addition of enzymatically degradable components or biodegradable polymers with collagen scaffolds to enable controlled degradation while supporting tissue cell infiltration and tissue regeneration. Mimicking the natural ECM composition of the meniscus, including GAGs and other ECM components, could further enhance scaffold performance by influencing its mechanical properties and degradation behavior. To guide future scaffold design, long‐term in vivo studies are essential for evaluating the degradation dynamics of collagen scaffolds in the complex environment of meniscal repair.

Successful meniscal regeneration depends on the coordinated delivery of biochemical cues and reparative cells. Among bioactive factors, transforming growth factor beta (TGF‐β), particularly TGF‐β3, remains one of the most potent drivers of fibrochondrogenic differentiation, stimulating Type I/II collagen synthesis and enhancing integration strength when released in a sustained manner [116]. Insulin‐like growth factor‐1 (IGF‐1) and platelet‐derived growth factor (PDGF) promote cell proliferation and matrix production, while connective tissue growth factor (CTGF) supports fibrocartilage formation and matrix remodeling [117]. Vascular endothelial growth factor (VEGF) plays a dual role, facilitating angiogenesis at the vascularized red–red periphery but requiring careful spatial control to prevent abnormal vascular invasion into the white–white zone [118]. These growth factors collectively orchestrate the distinct regenerative environments required across meniscal zones.

To translate these molecular cues into effective regeneration, controlled and localized release is essential. Several strategies have shown promise. Polymer‐based microspheres and nanoparticles, particularly poly(lactic‐co‐glycolic acid) (PLGA) carriers, can be embedded within collagen matrices to enable sustained release over several weeks with adjustable kinetics determined by polymer composition and molecular weight [119]. Affinity‐based delivery systems using heparin‐modified collagen or decellularized ECM prolong growth‐factor retention through reversible binding interactions, preserving bioactivity and minimizing burst release [120]. In parallel, fiber‐based release systems, such as electrospun or melt electrowritten collagen–PCL hybrids, allow growth factors to be co‐loaded within aligned fibers, coupling biochemical signaling with topographical guidance for cell organization [121].

Microencapsulation technologies further enhance spatial control. Microspheres or microgels composed of PLGA, gelatin, or alginate can be distributed selectively within scaffold regions, enabling zonal delivery that mirrors native meniscal physiology. For instance, VEGF‐loaded capsules can be confined to the peripheral layer to promote vascularization, while inner zones incorporate reservoirs of TGF‐β3 or IGF‐1 to induce chondrogenic differentiation [122, 123]. Such compartmentalized delivery achieves a spatial gradient of biochemical stimuli analogous to the natural red–red to white–white transition and reduces the risk of excessive angiogenesis in the central region.

The incorporation of MSCs provides complementary regenerative potential. Bone marrow–, adipose‐, and synovium‐derived MSCs are the most extensively studied sources, valued for their paracrine signaling and ability to differentiate toward fibrochondrocytic phenotypes [80]. Optimizing MSC integration involves preconditioning with TGF‐β3 or hypoxia to prime fibrochondrogenic gene expression, three‐dimensional spheroid culture to enhance matrix deposition, and mechanical prestimulation on anisotropic scaffolds to align ECM production [124]. Co‐delivery of MSCs with sustained‐release systems ensures local trophic support and mitigates cell loss after implantation. Early translational studies have demonstrated improved matrix organization and integration strength when MSCs are combined with collagen scaffolds containing slow‐release TGF‐β3 depots [101].

Future scaffold systems are likely to combine multifactor release and cell‐based delivery within architecturally optimized frameworks. Dual‐compartment scaffolds can house VEGF‐releasing hydrogels in the outer ring and TGF‐β3/IGF‐1‐loaded microcapsules in the inner zone, recapitulating both vascular and avascular environments. Concurrently, embedding MSCs within aligned collagen or PCL–collagen fibers allows mechanical and biochemical signals to act synergistically to guide maturation. Emerging work on gene‐activated bioinks further suggests that sustained, in situ growth‐factor expression may replace exogenous dosing, offering a stable and self‐regulating regenerative environment [125].

Overall, integrating spatially controlled growth‐factor release with MSC‐based bioactivity represents a powerful approach to achieve durable, zonal meniscal regeneration. By uniting controlled pharmacokinetics with biologically responsive cell populations, next‐generation collagen scaffolds can more faithfully reproduce the biochemical and cellular gradients of the native meniscus and support stable, long‐term functional recovery [101].

### 7.3. Sustainable Sourcing and Green Chemistry

Recent advancements in tissue engineering have demonstrated growing emphasis on sustainable and ecofriendly approaches, with researchers increasingly focusing on utilizing natural‐based, sustainable materials for bioscaffolds. Collagen‐based scaffolds, when combined with biopolymers such as chitosan [126], alginate [127], silk fibroin [128], gelatin [129], and starch [130], present promising opportunities for improving scaffold properties. These materials not only contribute to sustainability but also exhibit superior biocompatibility, biodegradability, and minimal toxicity [131]. Additionally, aligning with the principles of sustainability, collagen sources derived from food industry by‐products—such as bovine hide offcuts [132], pig skin, chicken skin, eggshells [133], sheep legs [134], and fish scales [135]—provide an eco‐conscious and cost‐effective alternative for scaffold production. Future research must prioritize the development of green chemistry protocols that utilize biodegradable, nontoxic materials and environmentally friendly fabrication processes.

As our understanding of meniscal injury types and repair mechanisms expands, it will guide the design and structural optimization of artificial meniscus materials. Future scaffolds must not only mimic the mechanical and structural properties of native meniscus but also incorporate bioactive features that enhance integration and regeneration. By addressing these challenges, the field of meniscus tissue engineering is expected to achieve breakthroughs that will bring new hope to patients suffering from meniscal injuries.

Moving forward, the integration of interdisciplinary approaches, combining advances in biomaterial science, biofabrication, cell biology, and clinical expertise, will pave the way for the next generation of collagen‐based meniscus scaffolds. These innovations have the potential to improve regenerative medicine and the quality of life for millions of patients worldwide.

## 8. Conclusions

This review has explored the key aspects of meniscal injuries and the potential role of collagen scaffolds for effective repair. By establishing the prevalence and clinical significance of meniscal injuries, it emphasizes that successful repair requires scaffolds capable of replicating both its anisotropic architecture and zonal composition. The detailed examination of meniscal anatomy and function reinforces the critical role of a thorough understanding in guiding the design of effective scaffold‐based treatments.

The discussion extends to fundamental principles of tissue engineering, with a specific focus on collagen scaffolds as essential tools for encouraging tissue regeneration. The mechanical and biological properties of these scaffolds are analyzed, demonstrating their potential to mimic the natural meniscus and support cellular activities essential for healing. Advanced fabrication techniques, such as freeze‐drying, MEW, and multimaterial 3D printing, now enable controlled fiber alignment and compositional gradients that approach the native red–red to white–white zonation.

Future research should focus on integrating anisotropic architecture with spatiotemporally controlled bioactive delivery, achieving synchronized remodeling and mechanical maturation. Through the convergence of advanced fabrication, mechanobiological modulation, and biologically intelligent design, next‐generation collagen scaffolds hold the promise to achieve durable functional repair and ultimately restore the complex biomechanics of the human meniscus. As research continues to evolve, the insights gained from this review will be useful to guide future innovations and clinical practices aimed at restoring knee health and function.

## Conflicts of Interest

The authors declare no conflicts of interest.

## Funding

This work was supported by the School of Engineering, University of Leicester, UK, and China Scholarship Council (CSC), 202206060010.

## Data Availability

Data sharing is not applicable to this article as no datasets were generated or analyzed during the current study.
